# Numerical Simulation of Atmospheric Pollutant Dispersion on Campus: Impacts of Wind Environment and Newly Constructed Buildings’ Height

**DOI:** 10.3390/jox16030105

**Published:** 2026-06-04

**Authors:** Chongxi Liao, Luxin Ren, Lulu Xu, Renjie Zhao, Baocong Zhao, Sihao Lin, Ting Zhang, Yijie Zhuang, Yanpeng Gao, Yuemeng Ji

**Affiliations:** 1 Guangdong-Hong Kong-Macao Joint Laboratory for Contaminants Exposure and Health, Guangdong Key Laboratory of Environmental Catalysis and Health Risk Control, Institute of Environmental Health and Pollution Control, Guangdong University of Technology, Guangzhou 510006, China; chongxi_liao@163.com (C.L.); renluxin_2019@163.com (L.R.); xululuwow@163.com (L.X.); z_ranchay@163.com (R.Z.); zbc201011@163.com (B.Z.); lsh0777@163.com (S.L.); zyj7933@gdut.edu.cn (Y.Z.); 2 Guangdong Basic Research Center of Excellence for Ecological Security and Green Development, Key Laboratory of City Cluster Environmental Safety and Green Development of the Ministry of Education, School of Environmental Science and Engineering, Guangdong University of Technology, Guangzhou 510006, China; 3 College of Civil Engineering, Liaoning Technical University, Fuxin 123000, China; memchem@163.com

**Keywords:** air pollutants, dispersion, toluene, VOC pollution, computational fluid dynamics

## Abstract

Toluene, as a common organic solvent in academic laboratories in university campuses, poses potential exposure concerns to students and staff in university campuses. Hence, by using a computational fluid dynamics simulation, we investigated the dispersion characteristics of toluene at a campus in Guangzhou under meteorological conditions and the impact of newly constructed buildings on toluene concentrations. The numerical simulation results reveal that toluene is readily accumulated in the free movement area under the prevailing east wind, in the administrative area under the prevailing north-northeast wind, and in the teaching area under the prevailing south wind. Therein, the teaching buildings (TB3–TB6) possess the highest average concentration of toluene compared with other functional areas. In the presence of newly constructed buildings, the toluene concentrations are decreased under the south-southeast wind but are aggravated under the southeast wind. As the height increases, under south-southeast winds, the merging of vortex structures continuously reduces toluene concentrations at TB3 and TB4 and the expansion of the wake region rebounds the toluene pollution at TB5 and TB6; under southeast winds, the expanding vertical vortex structures aggravate toluene pollution at TB3 and TB5 but attenuate toluene pollution at TB4 and TB6. Our results reveal that the teaching areas of the target campus represent a critical zone for potential student exposure during summer and require particular attention. This study provides new insights into the coupled effects of prevailing wind conditions and campus morphology on VOC dispersion characteristics and improves the understanding of airflow pollutant interactions in complex campus environments.

## 1. Introduction

Volatile organic compounds (VOCs) are a major component of hazardous air pollutants that pose potential exposure concerns to humans and affect indoor and outdoor air quality. The transport of VOCs leads to accumulations in certain areas, creating health risks to public health [1], and thus VOC dispersion near and around buildings is an important environmental problem. A higher health risk of school-aged children is enhanced by the accumulation of VOCs relative to adults [2]. However, an accurate prediction of VOC dispersion remains challenging owing to the complex interactions between atmospheric flow and building-induced airflow [3]. Therefore, it is essential to investigate the dispersion characteristics of VOCs to identify potential high-exposure areas, thereby alleviating exposure concerns in densely populated environments.

Meteorological conditions are the critical factors for atmospheric transport, with complex wind conditions playing a significant role in the dispersion of air pollutants [4,5]. A recent study has revealed that the pollution of PM_2.5_ and O_3_ is aggravated by the high temperatures and lower wind speeds [6]. It indicates that there is a significant correlation between meteorological conditions and air quality. Many studies focus on the meteorological parameters, such as the incoming wind speed and wind direction, atmospheric stability, and traffic-induced turbulence, that affect the vertical flow mixing and pollutant transport [7,8,9,10,11,12,13]. Yang et al. (2020) have pointed out that the dynamic retention and transport of air pollutants is governed by wind speed and direction [14]. A numerical simulation has found that the concentration of traffic pollutants in the street canyon is greatly reduced by the oblique wind directions compared with the perpendicular wind directions [15]. A previous study has shown that wind speed determines whether pollutants disperse vertically or spread horizontally, which significantly affects the spatial dispersion of traffic pollutants [16]. Handhayani (2023) revealed that the concentration of sulfur dioxide is affected by wind speed, and the number of unhealthy days rises with the average wind speed decreasing [17]. Hence, it is necessary to evaluate the regional dispersion characteristics of air pollution under distinct wind conditions and the impact of wind conditions on the dispersion of VOCs.

Furthermore, some studies have shown that the dispersion of pollutants is regulated by building geometry and urban morphology [18,19,20,21]. A study has found that there is a significant influence of building morphology and configuration on ventilation efficiency, thereby reducing virus transmission risks [22]. A recent study has revealed that PM_2.5_ concentrations are increased via street canyons composed of low-rise or multi-story buildings [23]. Therefore, understanding the influence of building geometry and layouts on pollutant dispersion in local regions is crucial for assessing air quality under specific meteorological conditions.

On the other hand, VOCs, as the major pollutants at university campuses, are emitted from laboratory exhaust stacks, with the concentrations significantly exceeding background levels [24]. A previous study has pointed out that chemical vapors from organic solvent evaporation account for more than 50% of the total VOCs detected within the campus environment [25]. In the campus environment, particles and gaseous pollutants are readily accumulated in leeward zones and at specific heights [26]. University campuses, as primary areas for education and scientific research, are characterized by complex building configurations and high population density. However, previous CFD studies mainly focused on the influences of idealized urban street canyons on pollutant dispersion, while studies on laboratory-emitted VOC dispersion in realistic university campuses remain limited. In particular, the combined effects of prevailing wind conditions and newly constructed buildings with varying heights on airflow structures and pollutant accumulation in complex campus environments are still poorly understood. Therefore, it is necessary to further investigate the interactions among wind direction, campus morphology, and VOC dispersion characteristics in realistic university environments.

In this work, we investigated the dispersion characteristics of the typical VOCs in the target university campus and their impact on various functional areas by using computational fluid dynamics simulation. Herein, toluene is selected as a representative VOC because it is a major constituent of common organic solvents in university laboratories and is frequently associated with laboratory exhaust emissions [25]. Therefore, its dispersion characteristics can provide useful information for understanding solvent-related VOC transport and accumulation in university campuses. The dispersion characteristics of toluene under the different prevailing wind directions were assessed according to the annual meteorological conditions in Guangzhou. The effects of newly constructed buildings with different heights on toluene concentrations were explored, and the effect of building heights on the dispersion of toluene is discussed. The impact of the toluene dispersion on different functional areas on campus is assessed in different months.

## 2. Materials and Methods

### 2.1. Turbulence Model Selection and Governing Equations

The choice of turbulence model is important for campus-scale pollutant dispersion simulations. In this study, the standard k-ε model was employed as the Reynolds-averaged Navier-Stokes (RANS)-based turbulence model. Compared with a large eddy simulation (LES), RANS is computationally more efficient and is therefore more suitable for campus-scale urban environments involving multiple wind directions and building height scenarios, although LES can better resolve transient turbulent mixing and instantaneous vortex structures [27,28,29]. Among RANS turbulence closures, the standard k-ε model has been widely applied and validated in outdoor and urban pollutant dispersion simulations. Zheng and Yang (2021) reported that the standard k-ε model achieved the best agreement with wind-tunnel measurements among five tested RANS models for street canyon flow and pollutant dispersion [30]. Cui et al. (2016) showed its good performance for urban flow and pollutant transfer under neutral to weakly buoyant conditions [31].

The CFD simulations were conducted by ANSYS-Fluent 2021 R1 (version 2021 R1; ANSYS Inc., Canonsburg, PA, USA) to resolve the air velocity and the pollutant concentration fields. We conducted the calculation based on the RANS conservation equations of mass, momentum, and energy for the incompressible turbulent flow. The governing equations are as follows:

Continuity equation:

(1) $$\frac{\partial u_{i}}{\partial x_{i}}=0$$

Momentum equation:

(2) $$\frac{\partial \rho \left(u_{i}u_{j}\right)}{\partial x_{i}}=-\frac{\partial p}{\partial x_{j}}+\frac{\partial }{\partial x_{i}}\left[\left(\mu +\mu_{t}\right)\left(\frac{\partial u_{i}}{\partial x_{j}}+\frac{\partial u_{j}}{\partial x_{i}}\right)\right]+S_{B}$$

Energy equation:

(3) $$\frac{\partial \left(u_{i}T\right)}{\partial x_{i}}=\frac{1}{C_{p}}\frac{\partial }{\partial x_{i}}\left[\frac{\lambda }{\rho }\frac{\partial T}{\partial x_{i}}-C_{p}\bar{u_{i}^{’}T^{’}}\right]$$

where *u_i_* and *u_j_* denote the velocity components in the *i*-direction and *j*-direction (*i*, *j* = 1, 2, 3, corresponding to *x*, *y*, *z*), *x_i_* and *x_j_* represent the spatial coordinate. In Equations (1) and (2), *ρ*, *p*, *μ*, *μ_t_*, *S_B_* refers to air density, static pressure, dynamic viscosity, turbulent viscosity and source terms, respectively. *T*, *λ*, *C_p_* and $\bar{u_{i}^{’}T^{’}}$ in Equation (3) denote time-averaged temperature, thermal conductivity, specific heat capacity and turbulent heat flux term.

The turbulence kinetic energy and its rate of dissipation are obtained from the following transport Equations (4) and (5).

(4) $$\frac{\partial }{\partial t}\left(\rho k\right)+\frac{\partial }{\partial x_{i}}\left(\rho ku_{i}\right)=\frac{\partial }{\partial x_{j}}\left[\left(\mu +\frac{\mu_{t}}{\sigma_{\kappa }}\right)\frac{\partial k}{\partial x_{j}}\right]+G_{\kappa }+G_{b}-\rho \varepsilon -Y_{M}+S_{\kappa }$$

(5) $$\frac{\partial }{\partial t}\left(\rho \varepsilon \right)+\frac{\partial }{\partial x_{i}}\left(\rho \varepsilon u_{i}\right)=\frac{\partial }{\partial x_{j}}\left[\left(\mu +\frac{\mu_{t}}{\sigma_{\varepsilon }}\right)\frac{\partial \varepsilon }{\partial x_{j}}\right]+G_{1\varepsilon }\frac{\varepsilon }{k}\left(G_{\kappa }+G_{3\varepsilon }G_{b}\right)-G_{2\varepsilon }\rho \frac{\varepsilon^{2}}{k}+S_{\varepsilon }$$

where *G_k_* and *G_b_* represent the generation of turbulence kinetic energy from the mean velocity gradients and buoyancy, respectively. *Y_M_* represents the contribution of the fluctuating dilatation in compressible turbulence to the overall dissipation rate. On the basis of Launder’s experiment, *G*_1*ε*_, *G*_2*ε*_, and *G*_3*ε*_ are set to be 1.44, 1.92, and 0.09, respectively; *σ_k_* and *σ_ε_* are set to be 1.0 and 1.3 for turbulent Prandtl numbers of *k* and *ε*. *S_k_* and *S_ε_* denote user-defined source terms. In order to enhance the convergence probability, decrease the computational expenditure and achieve sufficient numerical precision; the semi-implicit method for pressure linked equations-consistent (SIMPLE) algorithm was selected. The first-order and second-order upwind schemes were used in the above-mentioned parameters calculation in Equations (1)–(5).

### 2.2. CFD Simulation Set-Up

The campus of Guangdong University of Technology was selected to be the study area, with spatial dimensions of 1200 m × 1000 m × 700 m in length, width, and height, respectively (Figure S1a). The emission source was determined based on previously measured VOC concentrations from Engineering Building 3 of Guangdong University of Technology. The measured TVOC concentration was 129.2 ppbv, among which aromatic hydrocarbons accounted for 21.8% and toluene accounted for 38.7% of the aromatic hydrocarbons, corresponding to a toluene concentration of 9.78 ppbv. In this study, the rooftop of Engineering Building 3 was simplified as an area source, and toluene was released with an emission rate of 6.54 × 10^−10^ kg/h based on the measured data [32]. The boundary conditions of the numerical model were defined as follows: (i) The top boundary of the studied domain in Figure S1a was regarded as the tropospheric boundary. (ii) The inflow and outflow boundaries of the domain were flexibly adjusted with the change of wind direction in the different months. (iii) No-slip wall boundary conditions, calculated by the standard wall function, were applied in the near-wall treatment. The pollutant source was set to be the mass flow inlet boundary. All the simulations were conducted at normal temperature and pressure (298.15 K and 101.325 kPa).

## 3. Results and Discussion

### 3.1. Grid Sensitivity Analysis and Validation of the Standard k-ε Turbulence Model

To optimize computational efficiency, grid sensitivity analysis, as a common practice for the RANS simulation, was carried out. Herein, we employ three kinds of grid conditions, i.e., Rough, Medium, and Fine grids, which were constructed by the same type of unstructured polyhedral cell and with different cell numbers (Table S1). As shown in Table S1, the total cell numbers in the Rough, Medium and Fine grids are 0.28 × 10^6^, 0.93 × 10^6^, and 1.78 × 10^6^, respectively, indicating that the Medium grid displays a better agreement with the Fine grid than the Rough grid. Therefore, considering the compromise between the computational time and accuracy, the scheme for the Medium grid was applied in the following study.

On the other hand, to ensure the reliability of airflow predictions in toluene dispersion, the applicability of the standard k-ε turbulence model in predicting airflow structures and gas phase pollutant dispersion around buildings was validated using experimental wind tunnel data. Since the experimental data for toluene were unavailable, a single-block building wind tunnel experiment involving ethylene was performed to validate the standard k-ε turbulence model used in this study [33]. Since wind tunnel data for toluene dispersion around buildings were not available, a wind tunnel experiment using ethylene as a tracer gas was adopted to validate the CFD model. Ethylene was not used as a physicochemical surrogate for toluene but as a tracer gas for evaluating airflow-controlled gas transport around buildings. This method is reasonable for outdoor campus scale conditions, where toluene dispersion is mainly controlled by wind-driven transport and building-induced turbulent mixing rather than molecular diffusion. This approach is also supported by previous wind tunnel studies in which SF_6_ was used as a tracer gas to evaluate airflow–controlled pollutant transport. Cui et al. (2016) used SF_6_ as a tracer gas in wind tunnel experiments and found that pollutant dilution was strongly controlled by flow structures, indicating that tracer gases are suitable for evaluating airflow-controlled pollutant transport in built environments [34]. The prime parameters in our validation were set as follows (Figure S2): (i) The wind speed at the roof height of the source is 4.2 m/s. (ii) The simulated building is 0.1 m (length) × 0.1 m (width) × 0.2 m (height). (iii) Ethylene as the tracer gas is emitted from a ground-level point source, which is located at 0.25 H_0_ away from the leeward side of the building (H_0_ = 0.2 m). The velocity and pollutant concentration at measurement points in the model are distributed on every line of the four planes, including y/H_0_ = 0, x/H_0_ = 0.25, x/H_0_ = 0.5 and z/H_0_ = 0.0625. The Reynolds number was greater than 3.7 × 10^4^, exceeding the minimum criterion of 1.5 × 10^4^ suggested by Meroney [35], indicating that gas-phase pollutant transport is primarily controlled by flow field structures rather than by molecular diffusion. Therefore, Reynolds-number independence was achieved, and the similarity requirement was satisfied. Herein, four main statistical indexes were used to assess the model performance, involving normalized mean square error (NMSE), fractional bias (FB), normalized mean bias (NMB), and the coefficient of determination (R^2^). Based on the above-mentioned indices, Table S3 presents the validation results of the standard k-ε model applicability for ethylene concentration. The model was considered acceptable if it met the following confidence intervals [36]:

NMSE < 3, −0.3 < FB < 0.3, −0.5 < NMB < 0.5, R^2^ < 1.

It is evident from Table S3 that the standard k-ε model showed acceptable agreement with the wind tunnel data, with NMSE = 0.1381, FB = 0.2066, NMB = 0.1873, and R^2^ = 0.9535. These values fall within the recommended acceptance criteria, indicating that the standard k-ε model can reasonably reproduce the main dispersion characteristics around buildings. Hence, the standard *k-ε* model is expected to provide a description of toluene dispersion in this study.

### 3.2. Dispersion Characteristics of Toluene in the Campus

**Figure 1:**
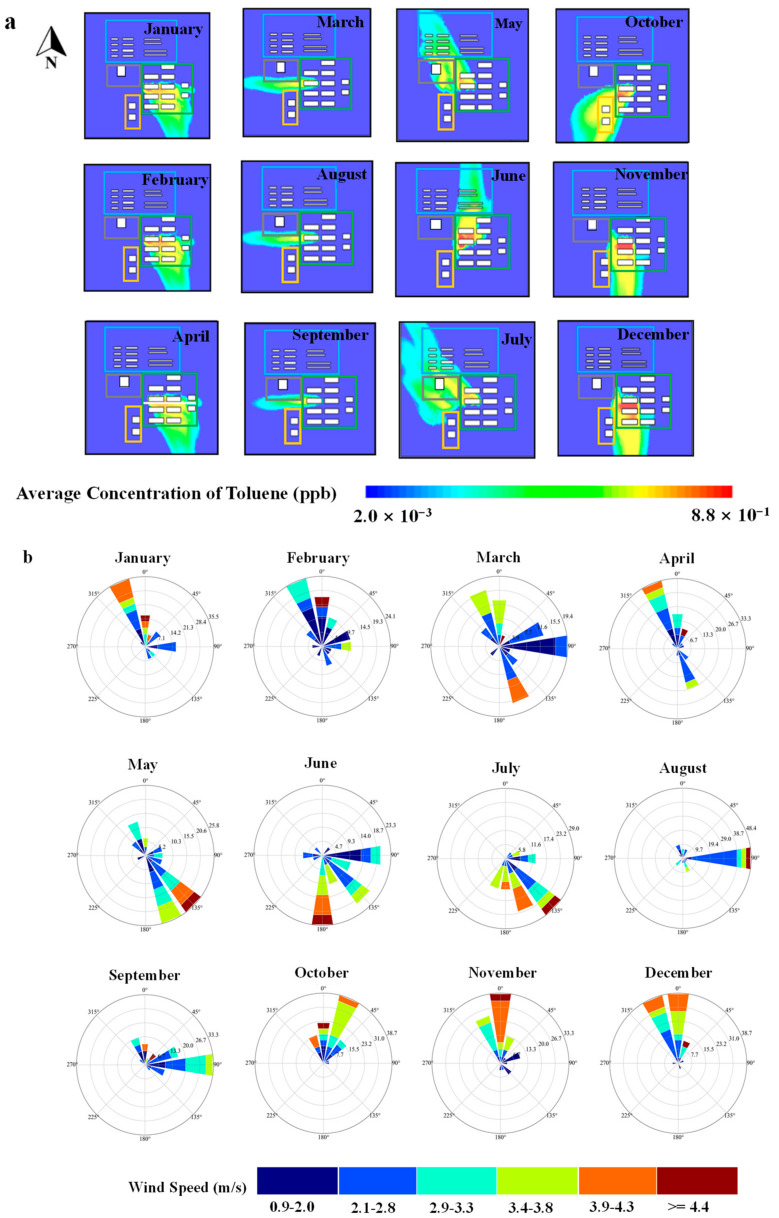
The annual characteristics of (**a**) the toluene dispersion pattern and (**b**) the wind rose for the university in Guangzhou from January to December. The concentration fields correspond to converged simulations.

**Figure 2:**
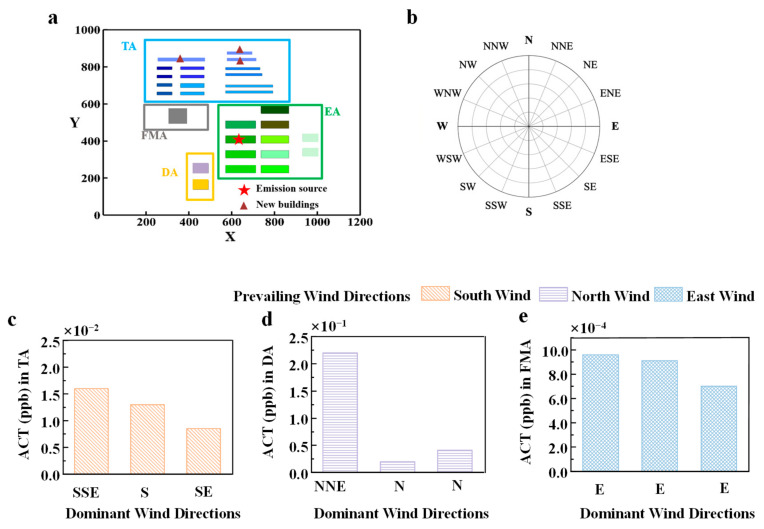
(**a**) The configurations of the campus functional areas and building layouts, (**b**) the wind rise of wind directions, and average concentration of toluene in (**c**) TA under the prevailing wind directions of south wind (SW), (**d**) DA under the prevailing wind directions of north wind (NW) and (**e**) FMA under the prevailing wind directions of east wind (EW). The concentration fields correspond to converged simulations.

We focus on the dispersion characteristics of toluene in the target university under the different wind directions, since the dispersion patterns of pollutants in Guangzhou are significantly affected by wind direction rather than by wind speed. In Guangzhou, there are three prevailing wind directions throughout the year, i.e., south wind (SW), north wind (NW), and east wind (EW). In this text, the dominant wind direction (DWD) of SW focuses on SSE, SE, and S, the DWD of NW focuses on NNW, NNE and N, and the DWD of EW is E according to the meteorological conditions in Guangzhou. Figure 1 shows the spatial dispersion of toluene and the annual wind rose in the study site. Figure 2 shows the average concentration of toluene (ACT) under three prevailing wind directions. In this study, ACT refers to the area weighted surface average of toluene concentration over the selected building surfaces, calculated from the converged concentration field under each simulated scenario. Before going further, it will be useful to divide the target university into three functional areas based on its functions (Figure S1b): (i) The teaching area (TA, marked with a blue box). (ii) The free movement area (FMA, marked with a gray box). (iii) The administrative area (DA, marked with a yellow box).

For the case of EW, the highest value of ACT is observed in FMA of 9.6 × 10^−4^ ppb, which is one to seven orders of magnitude higher than other areas. The spatial dispersion patterns reveal a trend of toluene transport from the source toward FMA, indicating that FMA is the main contaminated area under the prevailing east wind direction. For the case of NW, DA is the most severely contaminated area, with the highest value of ACT for 2.2 × 10^−1^ ppb, which is one to ten orders of magnitude higher than other areas under the NNE wind. OB1, which is located in the northern part of DA, exhibits the higher ACT value (2.8 × 10^−1^ ppb) compared with OB2, which lies in the southern part of DA (Table S4). It is attributed to the shorter duration of dilutive turbulent mixing around OB1 than OB2, corresponding to the shorter transmission distance of toluene from the source to OB1. However, it is observed from Figure 1a that DA is not affected by toluene under NNW and N winds. Therefore, FMA is the most contaminated building zone in EW, and it is necessary to pay more attention to the diverse impact of toluene pollution on DA under NNE wind.

As for the case of SW, the highest ACT occurs in TA with the value of 1.6 × 10^−2^ ppb, which is about one to five orders of magnitude higher than the other areas. Herein, we mainly investigate the dispersion characteristics of toluene for six buildings in TA in this section. As shown in Figure S1b, two buildings are located in the eastern part of TA, corresponding to TB1-TB2, and four buildings lie in the western part of TA, corresponding to TB3–TB6. The two buildings in the eastern part possess a lower ACT value relative to those in the western part under the SSE wind. For example, the lowest ACT value is observed at TB2 with 2.3 × 10^−7^ ppb (Table S4), which is five orders of magnitude lower than TB3 (7.0 × 10^−2^ ppb). It implies that TB3 was the most severely affected building zone under the SSE wind. Similarly, under the SE wind, the lowest value of ACT also occurs in the eastern part, while the highest one is observed in the western part. For example, the lowest ACT value is observed at TB2 with 1.1 × 10^−11^ ppb (Table S4), which is nine orders of magnitude lower than TB5 (2.4 × 10^−2^ ppb). By contrast, for the case of the S wind, toluene is mainly spread to the eastern part of TA, and the highest ACT is observed at TB1 of 3.5 × 10^−2^ ppb. Generally, toluene is primarily diffused to the western part of TA, i.e., TB3 and TB5, under SSE and SE winds, but is accumulated on the east side, i.e., TB1, under the S wind. Considering that TA is the center of the students’ activities, it is essential to pay attention to the potential exposure concerns for the students in TA, especially in the three building zones mentioned above. These results indicate that pollutant dispersion in a realistic campus layout is strongly dependent on the interaction between prevailing wind direction and building arrangement. Building on previous CFD studies of pollutant dispersion in street canyons and simplified building arrays, the present results further illustrate that wind directions can lead to different accumulation zones in the FMA, DA, and TA under different prevailing wind conditions.

### 3.3. Dispersion Characteristics of Toluene in the Presence of New Buildings

Recently, two building groups have been constructed in the eastern and western parts of TA, which will modify the local airflow and the concentration of toluene, thereby affecting potential exposure conditions. Therefore, it is essential to investigate the dispersion characteristics of toluene in TA in the presence of these newly constructed buildings (NCBs). In the following study, we focus on the toluene dispersion characteristics of toluene in TB3 to TB6, which exhibit a large concentration of toluene under SSE and SE winds, and evaluate the impacts of building height on the dispersion characteristics of toluene under SSE and SE wind conditions.

**Figure 3:**
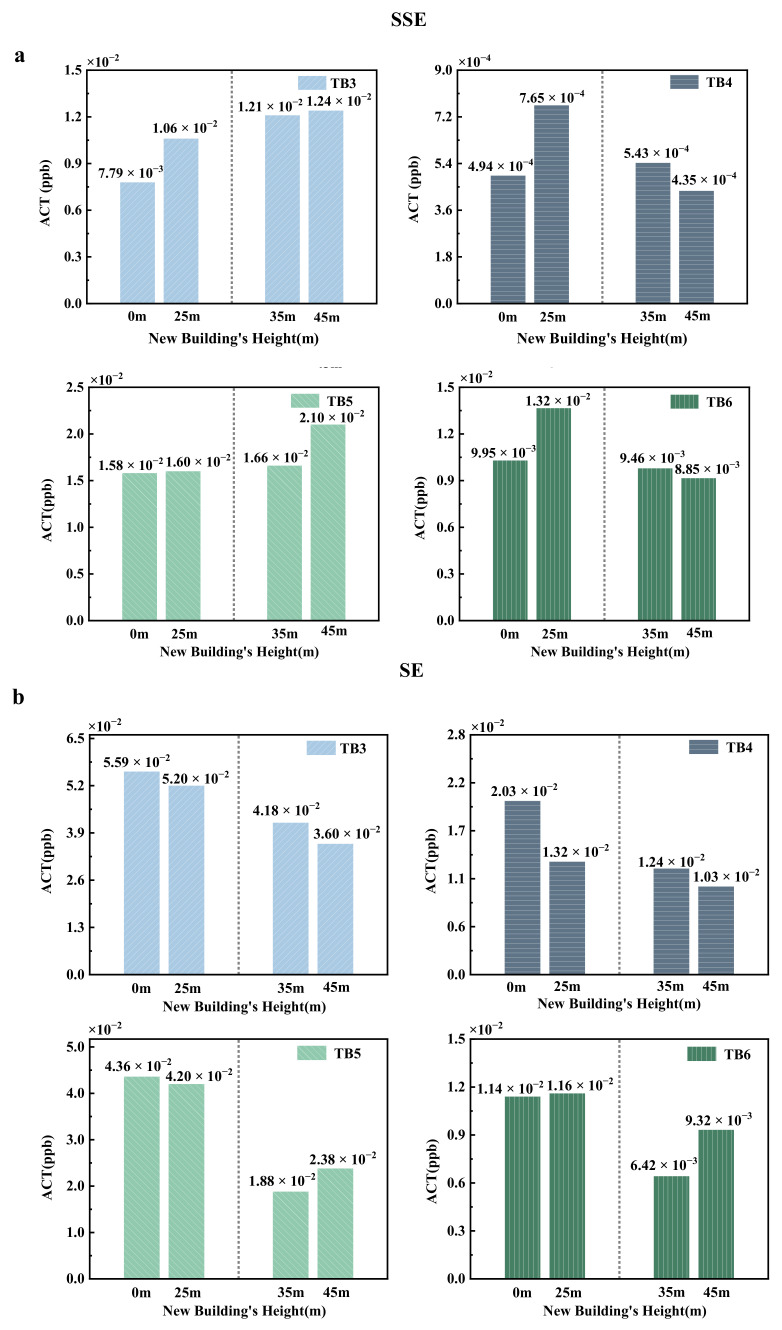
Average toluene concentration (ACT) at teaching buildings (TB3-TB6) under different heights of newly constructed buildings (0, 25, 35, and 45 m). Subfigure (**a**) shows results under south-southeast (SSE) wind, and subfigure (**b**) shows results under southeast (SE) wind.

Figure S3 depicts the dispersion patterns of toluene under the NCB heights of 25 m, and the corresponding ACT values are presented in Figure 3. Under the SSE wind, the ACT values are 5.20 × 10^−2^, 1.32 × 10^−2^, 4.20 × 10^−2^, and 1.16 × 10^−2^ ppb for TB3–TB6, respectively, indicating that the concentration of toluene decreases with distance downwind, dropping from TB3 and TB5 to the more distant TB4 and TB6. However, under the SE wind, the relatively highest ACT value occurs at TB5 with 1.60 × 10^−2^ ppb, and TB4 has the relatively lowest ACT value of 7.64 × 10^−4^ ppb. It is evident from Figure 3 that, compared with the case without NCBs, the presence of NCBs leads to a decrease in ACT values at TB3-TB6 under the SSE wind but an increase under the SE wind. For example, relative to the case without the NCBs, the ACT value at TB3 in the presence of NCBs is decreased by 6.98% under the SSE wind but is increased by 36.07% under the SE wind. It indicates that toluene pollution at TB3-TB6 is attenuated in the presence of NCBs under the SSE wind, while it is significantly aggravated under the SE wind.

**Figure 4:**
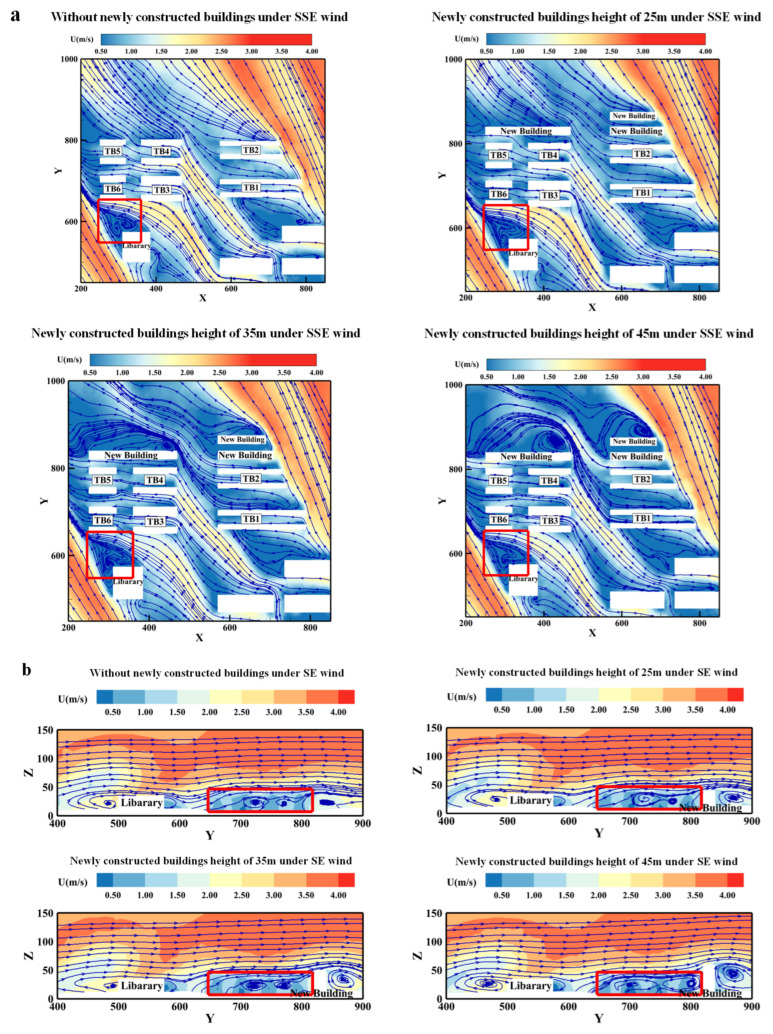
Velocity contours and streamlines in the teaching area (TA) under varying heights of newly constructed buildings (0, 25, 35, and 45 m). Subfigure (**a**) shows results under south-southeast (SSE) wind, and subfigure (**b**) shows results under southeast (SE) wind. The red frames represents the main vortex structure affecting the toluene concentration within the main affected area.

To further evaluate the impacts of building height on toluene dispersion patterns, the NCB heights of 35 m and 45 m are also investigated. The corresponding ACT values in TB3-TB6 under SSE and SE winds are also presented in Figure 3. Under the SSE wind, the ACT values at TB3-TB6 further decreased at the NCB height of 35 m (NCB_35m_), while a rebound trend of ACT values is observed at TB5 and TB6 at the NCB height of 45 m. For example, TB3 and TB6 still have the highest and lowest ACT values of 4.18 × 10^−2^ and 6.42 × 10^−3^ ppb at NCB_35m_, respectively, and, at NCB_45m_, the corresponding ACT values are 3.60 × 10^−2^ and 9.32 × 10^−3^ ppb. Compared with NCB_25m_, the highest concentration of toluene (TB3) is decreased by 19.61% at NCB_35m_ and 30.77% at NCB_45m_, while the lowest concentration of toluene (TB6) is increased by 45.17% at NCB_45m_. To explain the effects of building height on toluene dispersion, Figure 4 shows the airflow patterns around TB3-TB6 and the newly constructed buildings under SSE and SE wind conditions. Figure 4a is presented as a top view of the teaching area, while Figure 4b is shown as a vertical section. The arrows indicate the incoming wind direction, and the streamlines show the local airflow patterns. Analysis of flow patterns (Figure 4a) shows that the ambient wind speed at TB3 is accelerated by the merging of vortex structures in the northwest of the library as the building height increases, leading to the reduction in toluene concentration at TB3, while at TB6, there is a wake region behind NCB, which expands with the height of building increasing, resulting in the accumulation of toluene. Therefore, the dispersion of toluene is influenced by the height of NCB, while the concentration dispersion of toluene remains unchanged. However, under the SE wind, the highest concentration of toluene is located at TB5 at NCB_35m_ and NCB_45m_, with the ACT values of 1.66 × 10^−2^ and 2.10 × 10^−2^ ppb, which are more than one time larger than that at NCB_25m_. In contrast, the lowest ACT values at NCB_35m_ and NCB_45m_, which appear at TB4, are decreased by more than 30% compared with the case at NCB_25m_. It is attributable that the ventilation at TB4 is improved by the vertical vortex structures in front of NCB (Figure 4b), and the diameters of this structure expand with increasing building height, while TB5 is located at a relative distance from these expanding vortex structures, leading to toluene accumulation. In addition, it can be found that the highest concentration of toluene occurs at TB3 under the SSE wind but at TB5 under the SE wind. As the height of NCB increases, the concentration of toluene exhibits a decreasing trend at TB3 under the SSE wind but an increasing trend at TB5 under the SE wind. It indicates that differences in toluene concentration variations are induced by distinct wind directions. Hence, the dispersion characteristics of toluene are regulated by wind directions and the height of newly constructed buildings.

These flow field characteristics further suggest that the spatial differences in toluene concentration are governed by wind direction, ventilation and wake effects. Previous CFD studies have shown that pollutant dispersion in urban building arrays is strongly affected by wind direction, building morphology, and building height variation [23,37,38]. Changes in incoming wind direction can shift the dominant pollutant transport pathway by modifying the formation and location of recirculation and wake regions in street canyons and urban building blocks [15,39,40]. Building height variation can further reshape the vertical and horizontal exchange between the canopy layer and the overlying flow. Depending on the local configuration, the building height may enhance ventilation in some regions while creating stagnant or wake-dominated zones where pollutants accumulate [41,42,43]. Consistent with these findings, our results indicate that the combined effects of wind direction and building height changed the strength and position of vortical structures, altered the main routes of pollutant transport, and caused the accumulation of pollutants in the campus.

## 4. Conclusions and Atmospheric Implications

Organic solvent evaporation in university campuses may lead to localized VOC accumulation, which requires attention in campus air quality management, and thus the dispersion characteristics of organic solvents have garnered significant attention. Hence, we investigate the dispersion characteristics of toluene in a university campus and their impact on various functional areas using computational fluid dynamics simulation. The spatial dispersion patterns of toluene are determined by wind direction rather than wind speed in the target university campus under the meteorological conditions of Guangzhou. The simulation results demonstrate that prevailing wind direction plays a decisive role in determining the main affected functional zones on campus. Under different wind conditions, toluene accumulation may shift among the free movement area, the administrative area, and the teaching area. In particular, the teaching area is more sensitive to dominant south prevailing winds, suggesting that wind direction can change pollutant transport pathways and should be considered in campus air quality management. Furthermore, the newly constructed buildings were found to affect local airflow and toluene dispersion in the teaching area. Their effects depended on both building height and incoming wind direction. Under the SSE wind, the new buildings generally improved ventilation and reduced toluene accumulation around the teaching buildings. However, under the SE wind, the new buildings enhanced wake effects and promoted pollutant retention in teaching areas. From May to July, the dominant south wind direction enhances the accumulation of toluene and increases the potential exposure concerns in the teaching area. This suggests that the effects of new buildings on pollutant dispersion should be evaluated in combination with building height and prevailing wind direction, providing guidance for campus planning, building layout, and air quality management.

Several uncertainties remain due to the prescribed boundary conditions, simplified emission setting, and RANS turbulence modeling. In particular, the standard k-ε model may have limitations in resolving transient mixing, small-scale vortices, wake dynamics, and unsteady recirculation, which could affect the quantitative prediction of local concentration fields. Therefore, the results should be interpreted mainly as relative dispersion patterns and comparative trends under different wind direction and building height scenarios, rather than as direct exposure or health risk estimates. Future studies with more detailed meteorological and emission data would further improve the quantitative prediction of campus-scale VOC dispersion. Future studies should combine field measurements, variable emissions, detailed meteorology, non-isothermal boundary conditions, and higher-resolution turbulence models to improve the quantitative prediction of campus-scale VOC dispersion.

## Data Availability

The original contributions presented in this study are included in the article/Supplementary Material. Further inquiries can be directed to the corresponding author.

## Supplementary Materials

The following supporting information can be downloaded at: https://www.mdpi.com/article/10.3390/jox16030105/s1, Figure S1: The configurations of (a) the computational domain, (b) the heights and locations of newly constructed buildings in TA; Figure S2: The schematic illustrations of (a) the wind tunnel experiment, (b) the numerical model, and (c) the sampling point locations; Figure S3: Toluene diffusion patterns in TA under varying new building heights (0, 25, 35, and 45 m) in the prevailing south wind (SW); Table S1: Mesh parameters; Table S2: R^2^ assessment of average toluene concentration values (ppb) in Medium grid-*Fine* grid and Rough grid-Fine grid; Table S3: The characteristic index range of standard k-ε model verification and the verification results of ethylene concentration; Table S4: The values of average toluene concentration (ppb) around the building zones (TB1-TB6 and OB1-OB2) in TA and DA under the south (SW) and north (NW) prevailing wind directions, respectively.
