# Supplementary material for: Numerical Simulation of Atmospheric Pollutant Dispersion on Campus: Impacts of Wind Environment and Newly Constructed Buildings’ Height
Source: J Xenobiot. 2026 Jun 4;16(3):105. doi: 10.3390/jox16030105 (PMC13301529; doi:10.3390/jox16030105)
Supplement: Supplementary file 1 [file jox-16-00105-s001.zip › jox-4325138-supplementary.pdf]

Journal of *Xenobiotics*

MDPI

Article

Numerical Simulation of Atmospheric Pollutant Dispersion on Campus: Impacts of Wind Environment and Newly Constructed Buildings' Height

Chongxi Liao, Luxin Ren, Lulu Xu, Renjie Zhao, Baocong Zhao, Sihao Lin, Ting Zhang, Yijie Zhuang, Yanpeng Gao and Yuemeng Ji

1. Methods

The coefficient of determination is obtained from the following equation (1).

$$R^2 = 1 - \frac{\sum_{i=1}^{n}(y_i - \hat{y}_i)^2}{\sum_{i=1}^{n}(y_i - \bar{y})^2} \quad (1)$$

Where y_i denotes the observed value of the dependent variable for the i-th data point, $\hat{y}_i$ denotes the predicted value of the dependent variable for the i-th data point generated by the regression model, $\bar{y}$ denotes the mean value of all observed y_i, $\sum_{i=1}^{n}(y_i - \hat{y}_i)^2$ denotes the sum of squared residuals (SSR), quantifying the total discrepancy between observed and predicted values, $\sum_{i=1}^{n}(y_i - \bar{y})^2$ denotes the total sum of squares (SST), representing the total variability in the observed data relative to the mean. R^2 theoretically spans from $-\infty$ to 1, where values closer to 1 indicate that a larger proportion of the variance in the dependent variable is explained by the model, reflecting a stronger predictive capability.

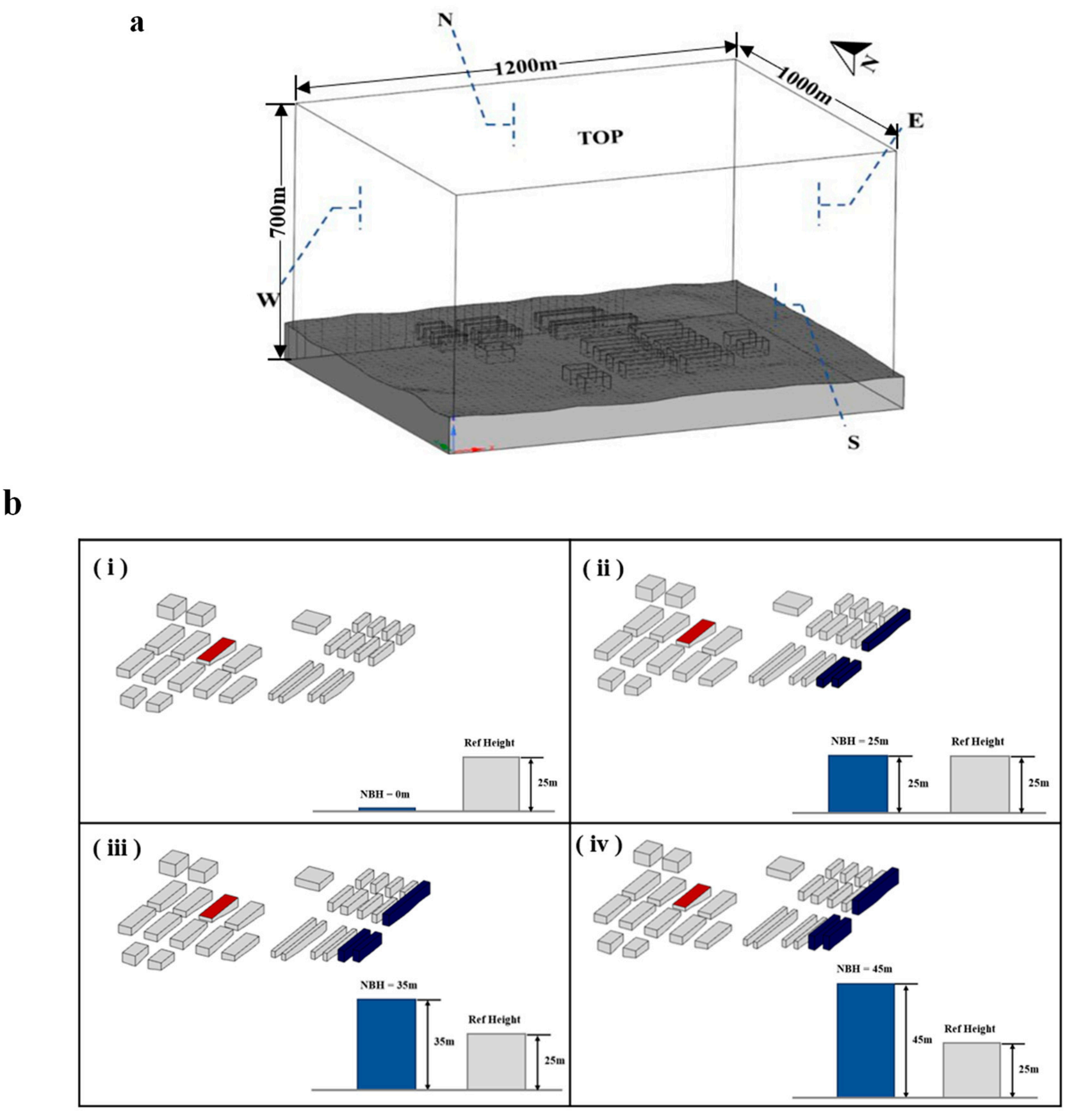

Figure S1. The configurations of (a) the computational domain, (b) the heights and locations of newly constructed buildings in TA.

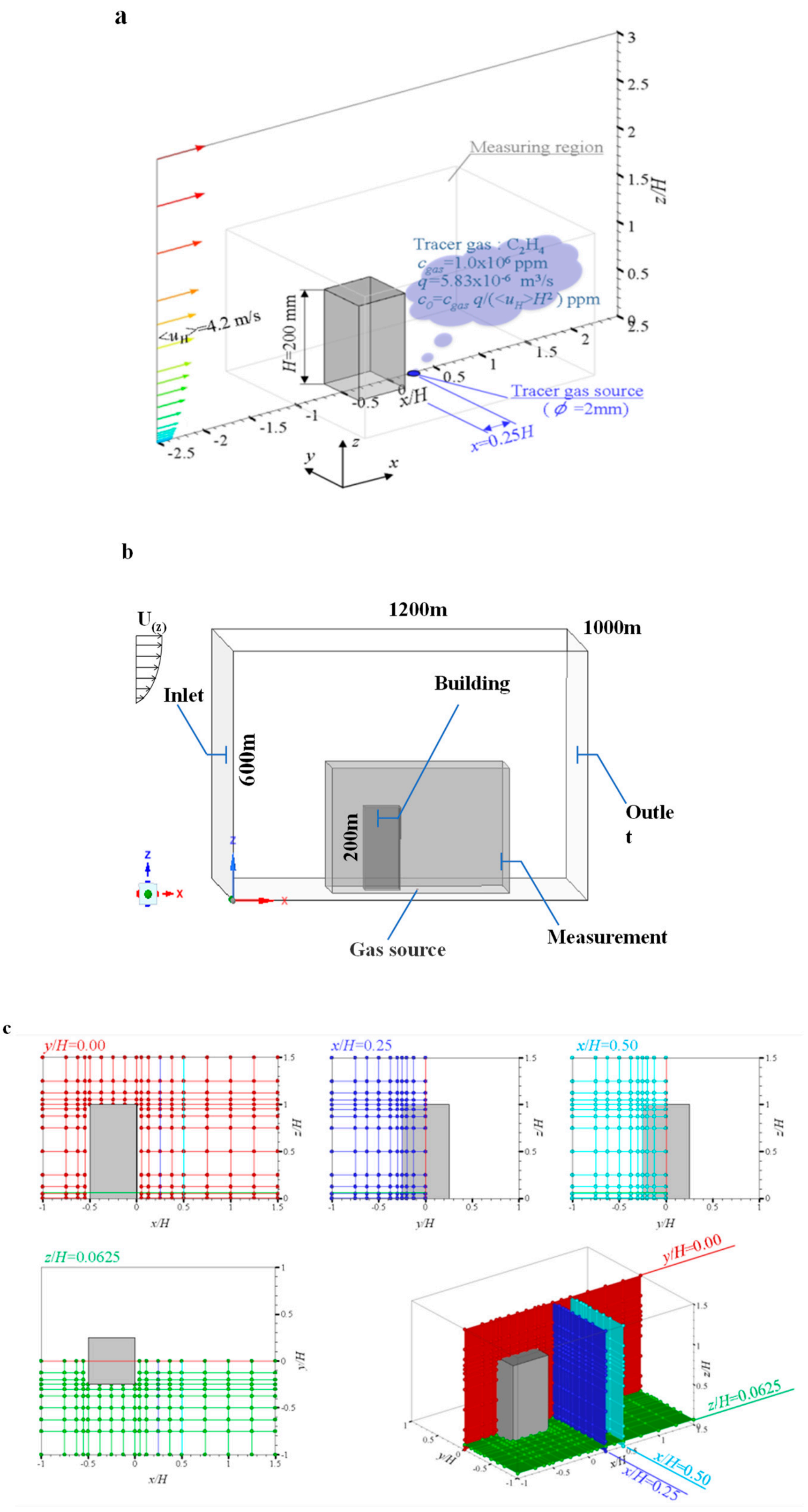

Figure S2. The schematic illustrations of (a) the wind tunnel experiment, (b) the numerical model, and (c) the sampling point locations.

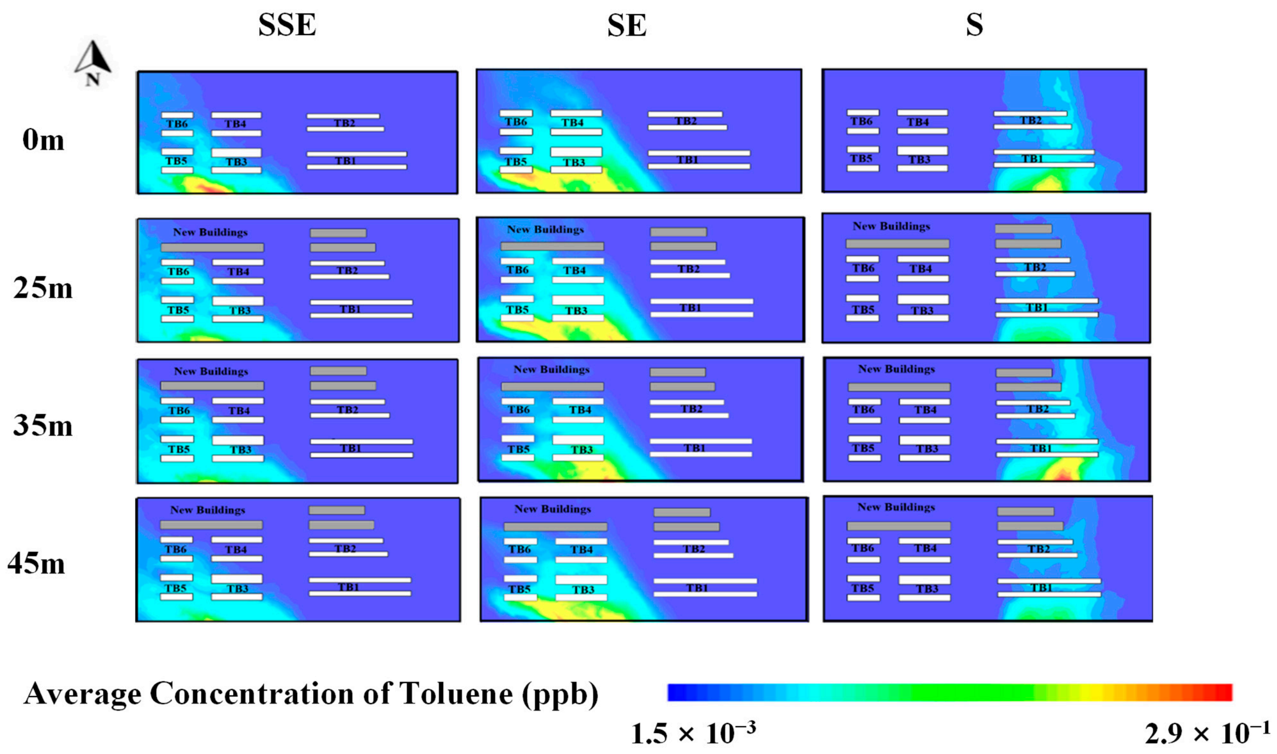

Figure S3. Toluene diffusion patterns in TA under varying new building heights (0, 25, 35, and 45m) in the prevailing south wind (SW).

Table S1 Mesh parameters.

Parameter	Rough	Medium	Fine
Number of grids	283310	930734	1786730
Average quality	0.83	0.84	0.85
Expansion rate	1.20	1.20	1.20

Table S2 R^2 assessment of average toluene concentration values (ppb) in *Medium* grid-*Fine* grid and *Rough* grid-*Fine* grid.

Contrast parameters	Acceptance criteria	$C_{Rough\text{-}Fine}$ (ppb)	$C_{Medium\text{-}Fine}$ (ppb)
Correlation coefficient（R^2）	1.0000	0.9032	0.9889

Table S3 The characteristic index range of standard k-ε model verification and the verification results of ethylene concentration.

Contrast parameters	Acceptance criteria	Concentration
Fractional mean bias (FB)	$-0.3 < FB < 0.3$	0.2066
Normalized mean-square error (NMSE)	$NMSE < 3$	0.1381
Normalized mean bias (NMB)	$-0.5 < NMB < 0.5$	0.1873
Correlation coefficient (R^2)	1	0.9535

Table S4 The values of average toluene concentration (ppb) around the building zones (TB1-TB6 and OB1-OB2) in TA and DA under the south (SW) and north (NW) prevailing wind directions, respectively.

Prevailing Wind Direction	Dominant Wind Direction	TA						DA	
		TB1	TB2	TB3	TB4	TB5	TB6	OB1	OB2
SW	SSE	3.7×10^{-4}	2.3×10^{-7}	7.0×10^{-2}	2.3×10^{-2}	6.6×10^{-2}	1.1×10^{-2}	-	-
	SE	1.8×10^{-9}	1.1×10^{-11}	1.1×10^{-2}	1.1×10^{-3}	2.4×10^{-2}	1.1×10^{-2}	-	-
	S	3.5×10^{-2}	2.0×10^{-2}	2.3×10^{-10}	1.8×10^{-10}	2.4×10^{-11}	3.8×10^{-11}	-	-
NW	NNE	-	-	-	-	-	-	2.8×10^{-1}	2.8×10^{-4}
	N	-	-	-	-	-	-	1.7×10^{-7}	1.0×10^{-7}
	N	-	-	-	-	-	-	5.4×10^{-8}	1.5×10^{-7}
